# Does treatment method matter? A meta-analysis of the past 20 years of research on therapeutic interventions for self-harm and suicidal ideation in adolescents

**DOI:** 10.1186/s40479-020-00123-9

**Published:** 2020-05-11

**Authors:** Oswald D. Kothgassner, Kealagh Robinson, Andreas Goreis, Dennis Ougrin, Paul L. Plener

**Affiliations:** 1 Department of Child and Adolescent Psychiatry, Medical University of Vienna, Vienna, Austria; 2 School of Psychology, Victoria University of Wellington, Wellington, New Zealand; 3 Department of Clinical and Health Psychology, Faculty of Psychology, University of Vienna, Vienna, Austria; 4 Outpatient Unit for Research, Teaching and Practice, Faculty of Psychology, University of Vienna, Vienna, Austria; 5 Department of Child and Adolescent Psychiatry, Institute of Psychiatry, Psychology and Neuroscience, King’s College London, London, UK; 6 Department of Child- and Adolescent Psychiatry and Psychotherapy, Medical University of Ulm, Ulm, Germany

**Keywords:** Self-harm, Suicidal ideation, Adolescence, Suicidal behaviour, Depression, Self-injury, Nonsuicidal self-injury, NSSI

## Abstract

**Background:**

Self-harm is a clinically relevant and prevalent behaviour which peaks in adolescence. Given the high prevalence of self-harm, the high levels of psychiatric comorbidity, and its role as a risk factor for suicide, delivering evidence-based care is critical.

**Methods:**

We conducted a systematic review and meta-analysis of the literature on treating self-harm in adolescents (12–19 years) published in the last 20 years, identifying 25 randomised controlled trials. We calculated the effect of treatment interventions relative to active control conditions in reducing self-harm, suicidal ideation and depressive symptoms.

**Results:**

Overall, treatment interventions fared slightly better than active controls in decreasing self-harm (*d* = 0.13, 95% CI 0.04–0.22, *p* = .004), suicidal ideation (*d* = 0.31, 95% CI 0.12–0.50, *p* = .001) and depressive symptoms (*d* = 0.22, 95% CI 0.07–0.38, *p* = .006). Subgroup analysis of specific therapies revealed moderate effects of DBT-A in reducing self-harm (*d* = 0.51, 95% CI 0.18–0.85, *p* = .002) and suicidal ideation (*d* = 0.48, 95% CI 0.17–0.80, *p* = .003), as well as moderate effects of family-centred therapy in the treating suicidal ideation (*d* = 0.58, 95% CI 0.01–1.15, *p* = .049).

**Conclusions:**

The findings of our meta-analysis indicate that, overall, currently available treatments are effective in treating self-harm, suicidal ideation, and depressive symptoms in adolescence. Although the treatment intervention conditions showed only small to moderate effects in comparison to active controls, these differences were statistically significant and are clinically important. Further research is needed to understand the reduction in self-harm within active controls, which may arise due to the natural course of self-harm, or the potential efficacy of treatment as usual and enhanced usual care. Given the significant reduction of self-harm in active control conditions, delivering effective care to a large number of adolescents with self-harm may require developing stepped-care models in clinical practice. Expensive and poorly available treatments should be targeted at young people who most need them.

Self-harm, defined as intentional self-poisoning or injury of oneself irrespective of suicidal motivation or intent [26], is a behaviour widespread among adolescents. A recent review of 172 adolescent community samples reported a mean lifetime prevalence rate of 16.9% (range: 4.1 to 39.3%), as well as a concerning trend to suggest that prevalence rates have increased in recent years [19]. Engagement in self-harm typically begins between 12 and 13 years old, peaks around 15 and 16 years old, and decreases in older adolescence and adulthood [19, 53, 60]. Thus, adolescence represents a key developmental period for self-harm prevention and intervention efforts [74].

Recent years have seen a debate about the nomenclature of self-harm. Whereas ‘self-harm’ includes both acts with or without suicidal intent, the term ‘Nonsuicidal Self-Injury’ (NSSI) describes the deliberate and direct destruction or alteration of body tissue in a manner that is socially unaccepted and that occurs *without* suicidal intent [1, 44], and therefore excludes suicidal attempts. Although the intention behind an act of self-injury is clinically relevant and of the utmost importance for clinical risk assessment [61, 72], empirical evidence to support a clear distinction between self-injury with and without suicidal intent is lacking. A systematic review comparing studies that used a self-harm definition to those using an NSSI definition failed to find significant differences in mean lifetime prevalence rates (16.1% versus 18.0% [54]), suggesting that in adolescent community samples this nomenclatory distinction is not evident.

Self-harm without suicidal intent also seems to play a role in the development of suicidal thoughts and behaviours. A growing body of evidence demonstrates that engaging in NSSI increases the risk of subsequent suicidal ideation, suicide attempts, and death by suicide [5, 7, 27, 63]. In particular, the interpersonal–psychological theory of suicidal behaviour argues that engaging in NSSI increases a person’s capability for suicide by creating habituation to the psychophysiological aversiveness of self-injury [35], with recent evidence that engaging in NSSI is one of the strongest predictors of transition from suicidal thoughts to suicide attempts among adolescents [45]. In the context of this ongoing investigation, ‘self-harm’ is a pragmatic umbrella term that encompasses both suicidal behaviours (suicidal ideation and attempts) as well as NSSI [8]. Given the clinical significance of any form of self-harm, effective treatment interventions for reducing self-harm behaviours and suicidal ideation are urgently needed, as is seen as a top-priority for interventions such as Dialectical Behaviour Therapy [46].

To date, several systematic reviews and meta-analyses have evaluated the evidence for effective psychosocial treatment interventions for adolescents who self-harm. Among these, outcome criteria were mixed, with reviews focusing either on suicidal behaviours, NSSI, or self-harm. A Cochrane review evaluating 11 trials for adolescents with self-harm highlighted the paucity of evidence for treatment interventions, with (apart from two comparisons) all the evidence for specific interventions built from a single trial [28]. Little evidence was found to support the efficacy of group therapy, while Mentalization-Based Therapy, Therapeutic Assessment, and Dialectical Behaviour Therapy for Adolescents (DBT-A) all warranted further evaluation [28].

Similarly, a systematic review of 29 treatment studies (including 18 RCTs) for suicidal and nonsuicidal self-injurious thoughts and behaviours (SITBs) in youth found that no intervention met the standard to be classified as a ‘well-established intervention’, and only six interventions could be classified as ‘probably efficacious’ [21]. A meta-analysis focusing on suicide attempts or self-harm in adolescents evaluated evidence from 19 RCTs, finding that, following intervention, intervention groups had a lower proportion of adolescents with self-harm compared to active controls [57]. However, when focusing specifically on nonsuicidal self-harm (excluding suicide attempts), there was no statistically significant difference between treatment as usual (TAU) and intervention groups, although intervention conditions showed a trend towards better effectiveness compared to TAU [57]. Taken together, these reviews highlight the need for greater empirical investigation into the treatment of self-harm, including replication of promising treatment interventions.

More recently, an update to the systematic review of treatment interventions for SITBs in youth [21] focused exclusively on RCTs, including the 9 new trials available since 2015 [20]. In light of this additional evidence, DBT-A now met the standard to be classified as a ‘well-established intervention’ for reducing both self-harm and suicidal ideation and was classified as ‘probably efficacious’ for reducing NSSI and suicide attempts. Across several different treatment interventions, certain elements seemed to increase treatment efficacy, such as a family-centred approach, the inclusion of skills-training, and (except for suicide attempts) longer treatment duration. However, very few independent replications of treatment interventions were identified [20]. This limitation of the current evidence-base was also highlighted in another recent systematic review on interventions for adolescents with suicide attempts or self-harm, which found that, across 21 studies, only DBT-A and Cognitive Behavioural Therapy (CBT) demonstrated independent replications of treatment efficacy [33]. Thus, all systematic reviews and meta-analyses on the treatment of adolescent self-harm conclude that; i) evidence for the efficacy of therapeutic interventions is weak, and ii) independent replication of treatment effects are critically needed.

However, neither of the recent systematic reviews conducted a meta-analysis, and so point estimates of therapeutic effects, as well as factors that moderate these estimates, are currently unknown. As the literature on therapeutic interventions for adolescent self-harm has increased since 2015, we decided to conduct a meta-analysis of all controlled studies and RCTs published in the last 20 years, including the 12 studies not included in the most recent meta-analysis [57]. We also conduct sub-group analyses to assess for the effect of specific therapy types in reducing self-harm behaviours, suicidal ideation, and depressive symptoms, in order to gain a more nuanced understanding of the differential effects of treatment efficacy. Given that many trials showed efficacy in reducing self-harm both in the intervention and in the active control groups, we also investigated the effects of active controls in reducing self-harm. Control treatments such as Treatment As Usual (TAU) or Enhanced Usual Care (EUC) typically include frequent but rather unstructured sessions that are exemplary of routine clinical care. This warrants a focus on these control treatments, in addition to the investigation into the efficacy of treatment interventions over and above routine clinical care.

## Method

### Search strategy and inclusion criteria

A search of Google Scholar and PubMed databases was conducted using the keywords “Self-harm”, “Self-injury”, “Suicidal behaviour”, “Suicidal ideation”, “Cutting”, “Suicide” AND “Adolescents”, “Therapy” OR “Intervention” from December 1999 until December 2019.

Studies were included in the meta-analysis if they reported a randomized controlled trial (RCT) comparing therapeutic interventions with treatment-as-usual, and reported outcomes for self-harm and/or suicidal ideation in adolescents aged 12 to 19. We included trials in which the majority of participants engaged in self-harm at least once, but studies in which only a minority (< 50%) of the sample had engaged in self-harm or suicidal behaviour, or study populations where the majority of participants had neurological or developmental disorders (e.g., autism) were excluded. We also excluded studies focusing solely on pharmacological treatments as well as uncontrolled studies (e.g., pre-post evaluations). No limitations on language or publication status were invoked.

**Fig. 1:**
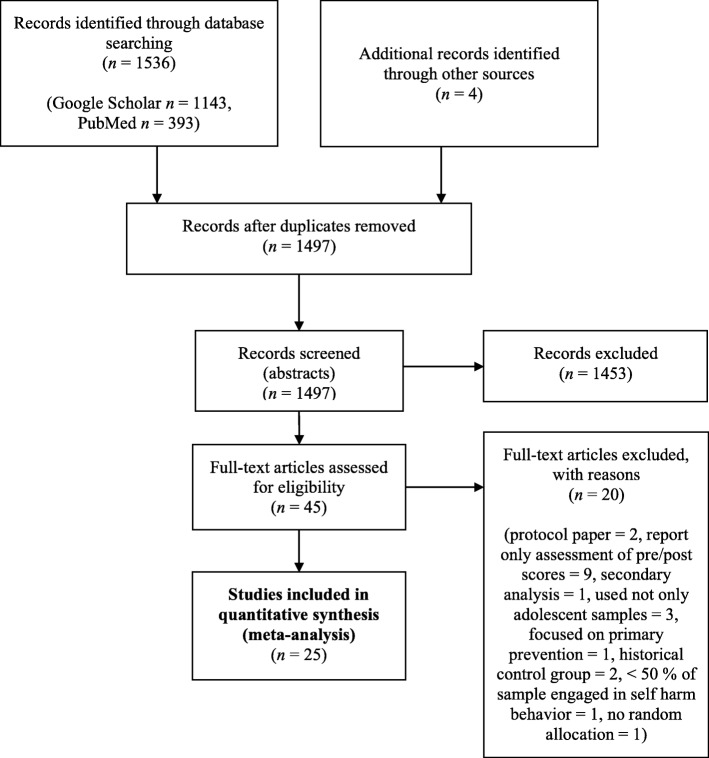
PRISMA flowchart showing the screening, exclusion, and inclusion criteria

We analysed the frequency of self-harm episodes and suicidal ideation as the primary outcome measures, with symptoms of depression as a secondary outcome measure, if reported. To analyse the effect of control interventions from pre- to post-measurement, we computed the standardized mean difference (Cohen’s *d*) based on the means and standard deviations [16] of self-harm, depressive symptoms, and suicidal ideation before and after the control interventions. The title, abstract, and main text of each study were examined, with the exclusion of documents occurring at each stage (see Fig. 1). The initial search generated 1536 results. The title and abstracts were screened for eligibility and full-text papers were obtained where necessary to evaluate inclusion. After screening, 25 studies––all peer-reviewed journal articles––were identified and included in our meta-analysis.

### Data extraction and analysis

Data from included studies were entered into a spreadsheet independently by two authors (ODK and KR) and differences were reviewed until consensus was reached. For each study included in the meta-analysis, we coded sample and intervention characteristics. The primary outcome was the standardized mean difference (Cohen’s *d*) in self-harm measured post-intervention. Secondary outcomes included Cohen’s *d* for depressive symptoms and suicidal ideation as assessed via various self-report measures in therapeutic intervention and active control conditions measured post-intervention. Mean, standard deviations and sample sizes were retrieved and inserted in a spreadsheet. If means or standard deviations were not reported in studies or supplemental materials, conversion via Revman [9] or formulas [4] were conducted. If episodes of self-harm were reported as proportions or odds ratios, they were transformed to Cohen’s *d* via formulas provided in Lipsey and Wilson [42].

We computed the standardized mean difference (Cohen’s *d*) between intervention and active controls as an indicator of the therapeutic intervention’s efficacy using the formula *d* = (*M*_Intervention_ –*M*_Control_)/*SD*_pooled_, with the respective means of measurements for the intervention and active controls. Effect size calculations and subsequent meta-analysis were then conducted with the metafor package for R [71], which automatically corrects Cohen’s *d* for the potential positive bias in small samples [30]. Following established conventions [10], an effect size of 0.20 was considered a small effect, 0.50 a medium effect, and 0.80 a large effect. Random effects models were applied to estimate aggregated effect sizes. Heterogeneity across study outcomes was reported with *I*^2^ values, where 25% indicates low, 50% moderate, and 75% high heterogeneity [32]. Egger’s regressions were conducted to estimate publication bias [67], with adjusted effect sizes calculated using trim-and-fill analyses and, based on funnel plot asymmetry, numbers of imputed missing studies [17]. Moderator analysis (meta-regression) was conducted to test whether treatment duration moderated the effect of the therapeutic interventions. All data and analysis code are available on the Open Science Framework (DOI:10.17605/OSF.IO/VR52S).

## Results

### Study characteristics

In total, 25 studies were identified (see Table 1), all of which were RCTs. The 25 studies comprised 2962 participants in total, of which 1515 received therapeutic interventions and 1447 received active control treatments.

**Table 1:** Characteristics of the 25 studies in the meta-analysis

Study	Outcome (Measures)	Age Group	% female	Therapeutic Intervention		Control Interventions		Dose / Duration	Drop-out	Eligibility Criteria (Recruitment Setting)
				***Treatment***	***n***	***Treatment***	***n***	***Means***	***n***	
[2•]	SI (SIQ-HS), D (BDI II)	15–18 yrs	0%	Dialectical Behaviour Therapy for Adolescents	10	Mode Deactivation Therapy	10	6 months	0	n. r. (residential care)
[3•]	SI (HASS)	10–18 yrs	69%	Family-based Cognitive Behaviour Therapy	89	Enhanced Usual Care	92	1 month	21	Presented to ED with a suicide attempt or SI (ED)
[6•]	SH (interview developed in house)	15–18 yrs	75%	Cognitive Analytic Therapy	35	Good Clinical Care	34	9 months	8	2–9 DSM-IV criteria for BPD, and during childhood ≥1 of any personality disorder criteria, disruptive behaviour symptoms, depressive symptoms, low socioeconomic status, and abuse or neglect (ED, primary care, family, school or self-referral)
[11•]	SH (SASII), SI (BSS)	11–17 yrs	89%	Family Therapy	268	Treatment as Usual	210	6 months	99	≥2 self-harm episodes and living with a primary caregiver willing to participate (mental health services)
[14•]	SI (SIQ-JR), D (BDI-II)	12–17 yrs	83%	Attachment-Based Family Therapy	35	Enhanced Usual Care	31	3 months	13	SI (SIQ-JR score ≥ 31) and depression (BDI-II ≥ 20) (primary care services and ED)
[13•]	SI (SIQ), D (BDI-II)	12–18 yrs	82%	Attachment-Based Family Therapy	66	Family-Enhanced Nondirective Supportive Therapy	63	4 months	14	SI (SIQ-JR ≥ 31) and depression (BDI-II ≥ 20) (ED, inpatient, mental health agencies, primary care services, schools, and community, or self-referral)
[15•]	SI (SIQ), D (CES-D)	12–17 yrs	82%	Skills-based Treatment	15	Supportive Relationship Treatment	16	6 months	8	Presented to ED or inpatient unit after a suicide attempt (ED and inpatient)
[18•]	SI (SIQ), D (RADS-2)	13–17 yrs	67%	Internet-based Cognitive Behaviour Therapy	19	Enhanced Usual Care	17	6 months	4	Suicide attempt within the past 3 months, or suicidal ideation (SIQ ≥ 41), alcohol or cannabis disorder, and lived with a caregiver willing to participate (inpatient)
[23•]	SH (SH interview), SI (SIQ), D (MFQ)	12–17 yrs	89%	Eclectic Group Therapy	181	Enhanced Usual Care	181	1.5 months	7	≥ 2 episodes of SH within the past 12 months (mental health services)
[29•]	SH (PHI), SI (SIQ), D (MFQ)	12–16 yrs	90%	Eclectic Group Therapy	34	Treatment as Usual	34	1 month	3	≥ 2episodes of self-harm in the past year, and ≥ 1 in the past 3 months (mental health services)
[31•]	SI (SIQ), D (RADS-2)	13–19 yrs	82%	Internet-based Cognitive Behaviour Therapy + Treatment as Usual	26	Treatment as Usual	24	2.5 months	11	Engaged with a well-being staff member, and any level of suicidal ideation in the last 4 weeks (secondary schools)
Kaess et al., 2019	SH (SITBI-G), D (BDI-II)	12–17 yrs	96%	Cognitive Behaviour Therapy (with elements of Dialectical Behaviour Therapy)	37	Treatment as Usual	37	4 months	2	NSSI ≥5 times in the past 6 months, and ≥ 1in the past month. (inpatient, outpatient, and community notices)
[38•]	SH (C-SSRS), SI (SIQ-R)	12–18 yrs	89%	As Safe As Possible Program (ASAP) + Treatment as Usual	34	Treatment as Usual	32	3–4 h. + TAU	6	Presented to inpatient with recent SI or a recent suicide attempt (inpatient)
[41•]	SH (CAFAS), SI (SIQ-JR), D (RADS)	12–17 yrs	68%	Psycho-educational social network intervention + Treatment as Usual	113	Treatment as Usual	123	~ 1 h. + TAU	102	Suicide attempt or SI within the past month and score of 20 or 30 on the self-harm subscale of the CAFAS (inpatient)
[40•]	SH (No. episodes) SI (SIQ-JR)	13–17 yrs	71%	Psychoeducational network intervention + Treatment as Usual	175	Treatment as Usual	171	~ 1 h. + TAU/telephone calls	92	Suicide attempt or SI within the past month (inpatient)
[39•]	SI (SIQ-JR), D (RADS-2)	14–19 yrs	80%	Motivational Interviewing	27	Enhanced Usual Care	22	~ 1 h. + TAU	3	SI, a recent suicide attempt, or both depression and substance abuse (ED)
[47•]	SH (No. episodes), SI (SIQ-JR)	12–18 yrs	95%	Dialectical Behaviour Therapy for Adolescents	86	Individual and Group Supportive Therapy	87	6 months	40	≥1 lifetime suicide attempts, suicidal ideation (≥24 SIQ-JR) in the past month, ≥3 lifetime SH, including 1 in the 12 weeks before screening, and ≥ 3 BPD criteria (ED, inpatient, outpatient, and community services)
[51•]	SH (No. episodes), SI (SIQ-JR), D (MADRS)	12–18 yrs	88%	Dialectical Behaviour Therapy for Adolescents	39	Enhanced Usual Care	38	4.75 months	0	≥3 self-harm episodes, ≥1 within past 16 weeks; ≥2 DSM-IV BPD criteria (or 1, with ≥2 subthreshold-level criteria) (outpatient)
[56•]	SH (No. episodes)	12–18 yrs	81%	Therapeutic Assessment	35	Assessment as usual	34	~ 7 h.	1	Not engaged with psychiatric services, presented to emergency services with SH, and referred for psychosocial assessment (ED or community services)
[59•]	SH (ASQ-R)	12–17 yrs	75%	Resourceful Adolescent-Parent Program	22	Treatment as Usual	18	1.5 months	8	≥1 episode of suicidal behaviour (SI, suicide attempt, or SH) within the past 2 months, living with 1+ parent, and a primary diagnosis of either major depression, posttraumatic stress disorder, or anxiety disorder (ED and primary care)
[64•]	SH (RTSHI), D (MFQ)	13–18 yrs	85%	Mentalization-based Therapy for Adolescents	40	Treatment as Usual	40	12 months	37	≥1 episode of SH within the past month (community mental health services and ED)
[65•]	SH (No. episodes)	14–19 yrs	88%	Emotion regulation group training (with elements of Cognitive Behaviour Therapy and Dialectical Behaviour Therapy)	14	Treatment as Usual	17	4.25 months	12	Mood instability due to increased reactivity, two forms of potentially self-damaging impulsivity, recurrent SH, inappropriate, intense anger, or difficulty controlling anger (outpatient)
[66•]	SH (No. episodes)	14–19 yrs	96%	Emotion regulation group training (with elements of Cognitive Behaviour Therapy and Dialectical Behaviour Therapy)	48	Treatment as Usual	49	4.25 months	9	≥2 DSM-IV BPD criteria (outpatient)
[68•]	SI (BSS), D (BDI-II)	12–18 yrs	66%	Intensive Interpersonal Psychotherapy-Adolescent	35	Treatment as Usual	38	1.5 months	3	Moderate-severe depression (BDI > 19), suicide ideation or previous suicidal attempt (BSS > 0), moderate-severe anxiety, or significant hopelessness in the past 2 weeks (secondary schools)
[73•]	SH (interview;), SI (SIQ), D (MFQ)	12–16 yrs	78%	Eclectic Group Therapy	32	Treatment as Usual	29	2 months	1	≥1 SH episode in the past year and referred following an episode of SH (mental health services)

*n. r.* not reported. Outcomes: *D* Depressive Symptoms, *SH* Self-harm, *SI* Suicidal Ideation, Measures: *ASQ-R* Adolescent Suicide Questionnaire-Revised, *BDI-II* Beck Depression Inventory-II, *BSI-D* Brief Symptom Inventory-Depression items, *BSI-SI* Brief Symptom Inventory-Suicidal Ideation items, *BSS* Beck Scale for Suicide Ideation, *CAFAS* Child and Adolescent Functional Assessment Scale, *CES-D* Center of Epidemiologic Studies-Depression Scale, *C-SSRS* Columbia Suicide Severity Rating Scale, *HASS* Harkavy-Asnis Suicide Scale, *MADRS* Montgomery–Åsberg Depression Rating Scale, *MFQ* Mood and Feelings Questionnaire, *PHI* Parasuicide History Interview, *RADS* Reynolds Adolescent Depression Scale, *RADS-2* Reynolds Adolescent Depression Scale, *RTSHI* Risk-Taking and Self-Harm Inventory, *SASII* Suicide Attempt Self-Injury Interview, *SIQ* Suicide Ideation Questionnaire, *SIQ-HS* Suicide Ideation Questionnaire High school Form, *SIQ-JR* Suicidal Ideation Questionnaire Junior, *SITBI-G* Self-Injurious Thoughts and Behaviours Interview-German version. Eligibility criteria: *BPD* Borderline Personality Disorder, *ED* Emergency Department

The most common therapeutic interventions investigated were either family-centred therapy (*n* = 5, 20%) or Group Therapy (*n* = 5, 20%), with the remainder of studies focusing on CBT-based interventions (*n* = 4, 16%), brief-interventions plus TAU (defined as up to 3 sessions in addition to treatment as usual, *n* = 4, 16%), DBT-A (*n* = 3, 12%), Mentalization-Based Treatment for adolescents (MBT-A; *n* = 1, 4%), Integrative Therapy (*n* = 1, 4%), Therapeutic Assessment (*n* = 1, 4%) and Cognitive Analytic Therapy (CAT, *n* = 1, 4%). The majority of active controls were comprised of Treatment as Usual (TAU, *n* = 13, 52%) and Enhanced Usual Care (EUC, *n* = 6, 24%). Other active controls used specific therapeutic interventions for comparison (*n* = 5, 20%) or specific routine care (Assessment as Usual, Hospital Care; *n* = 1, 4%).

### Control interventions

Given that TAU and EUC interventions which make up the majority of Control groups are exemplary models of routine clinical care, we first tested the efficacy of these control interventions in reducing self-harm, suicidal ideation, and depressive symptoms. Across 22 studies,^1^ participants receiving standard therapy (e.g., TAU, EUC) as part of active control groups showed moderate to large reductions from pre-to-post intervention in self-harm behaviour (*d* = 0.60, 95% CI 0.28–0.92, *p* < .001, *n =* 13), suicidal ideation (*d* = 0.87, 95% CI 0.35–1.39, *p* = .001, *n =* 11), and depressive symptoms (*d* = 0.51, 95% CI 0.07–0.94, *p* = .002, *n =* 14), suggesting that current routine clinical care is effective at improving patient outcomes.

### Efficacy of therapeutic interventions on self-harm

**Fig. 2:**
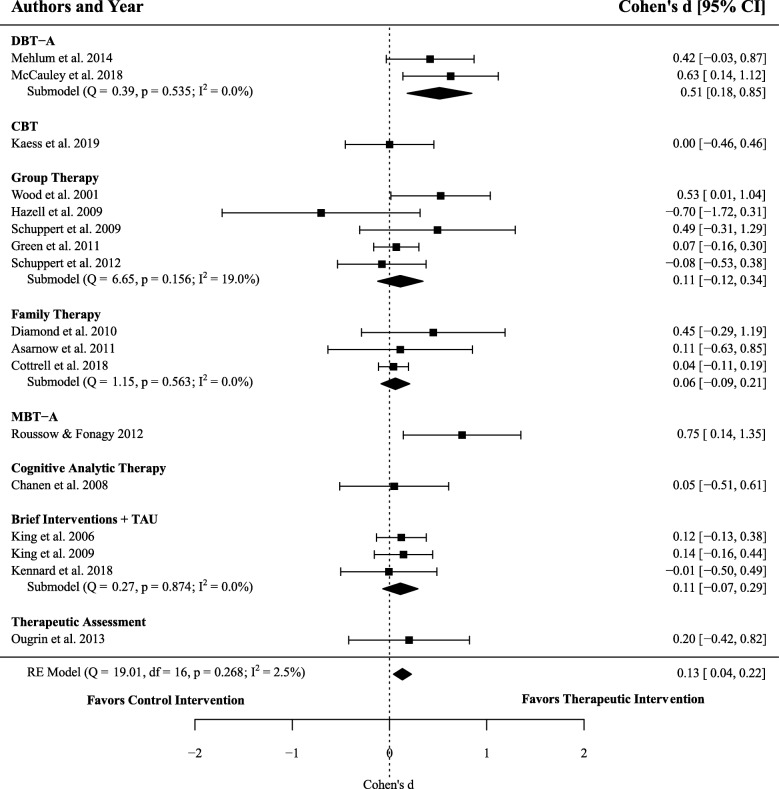
Forest plot of trials comparing the effect of therapeutic interventions and controls on self-harm. Note: Displays the standardized mean difference (Cohen’s *d*) in post-treatment self-harm, a positive effect size indicates that the outcome was in favour of therapeutic interventions. The average effect was calculated using a random-effects model

Seventeen effect sizes encompassing 2534 participants (1295 receiving therapeutic interventions and 1239 in active controls) were extracted in order to calculate the overall efficacy of therapeutic interventions, compared to active controls, for reducing self-harm behaviours. Meta-analysis resulted in a significant difference between groups (*d* = 0.13, 95% CI 0.04–0.22, *p* = .004) with low heterogeneity between studies (*I*^2^ = 2.51%). That is, therapeutic interventions showed a small, but statistically significant, improvement in reducing self-harm behaviours compared to control treatments. See Fig. 2 for a comparison of the effect of therapeutic interventions (compared to control) in reducing self-harm behaviours across different types of therapeutic treatments.

### Efficacy of therapeutic interventions on suicidal ideation

**Fig. 3:**
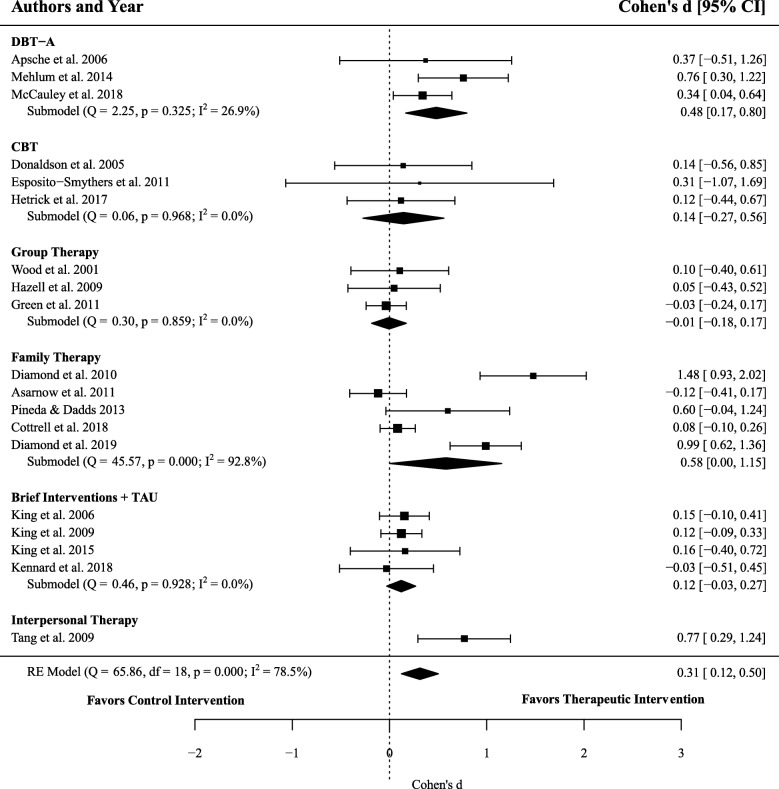
Forest plot of trials comparing the effect of therapeutic interventions and controls on suicidal ideation. Note: Displays the standardized mean difference (Cohen’s *d*) in post-treatment suicidal ideation, a positive effect size indicates that the outcome was in favour of therapeutic interventions. The average effect was calculated using a random-effects model

Nineteen effect sizes encompassing 2542 participants (1306 receiving interventions and 1236 in active controls) were extracted in order to calculate the efficacy of therapeutic interventions, compared to active controls, for reducing suicidal ideation. Meta-analysis resulted in a significant difference between groups (*d* = 0.31, 95% CI 0.12–0.50, *p* = .001), with high heterogeneity between studies (*I*^2^ = 78.51%) suggesting that therapeutic interventions were moderately more effective at reducing suicidal ideation than treatment in the active control groups. See Fig. 3 for a comparison of the effect of therapeutic interventions (compared to control) in reducing suicidal ideation across different types of therapeutic treatments.

### Efficacy of therapeutic interventions on depressive symptoms

**Fig. 4:**
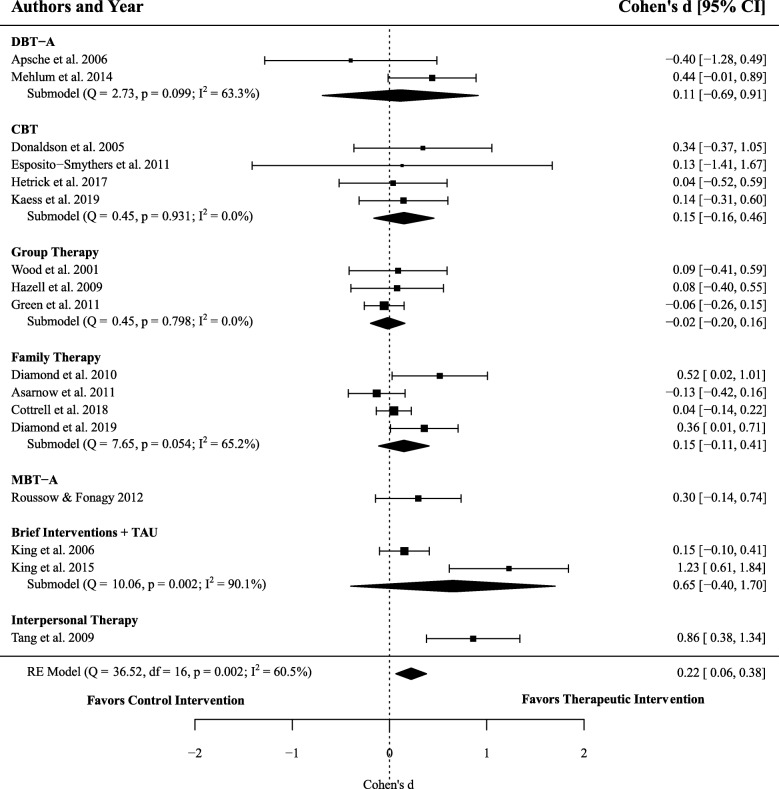
Forest plot of trials comparing the effect of therapeutic interventions and controls on symptoms of depression. Note: Displays the standardized mean difference (Cohen’s *d*) in post-treatment symptoms of depression, a positive effect size indicates that the outcome was in favour of therapeutic interventions. The average effect was calculated using a random-effects model

Seventeen effect sizes encompassing 2071 participants (1066 receiving therapeutic interventions, and 1005 in active controls) were extracted in order to calculate the overall efficacy of therapeutic interventions, compared to active controls, for reducing symptoms of depression. Meta-analysis resulted in a significant difference between groups (*d* = 0.22, 95% CI 0.07–0.38, *p* = .006), with moderate heterogeneity between studies (*I*^2^ = 56.70%). That is, therapeutic interventions were moderately more effective at reducing symptoms of depression compared to control treatments. See Fig. 4 for a comparison of the effect of therapeutic interventions (compared to control) in reducing depressive symptoms across different types of therapeutic treatments.

### Moderator analyses

To test whether treatment duration (in months) or proportion of young women in the overall sample (compared to young men) moderated the size of meta-analytic effect between therapeutic intervention and active controls, we conducted a multiple meta-regression (see Table 2 for coefficients). Analyses revealed none of these study aspects influenced the effect size of the difference between the therapeutic interventions and active controls for self-harm, suicidal ideation, or depressive symptom outcomes.

**Table 2:** Parameters of mixed-effects meta-regression on the efficacy of therapeutic interventions as compared to active controls in self-harm, depressive symptoms, and suicidal ideation

Predictors	Self-Harm (***n =*** 17)			Suicidal Ideation (***n =*** 19)			Depressive Symptoms (***n =*** 17)		
	*b*	*SE*	*p*	*b*	*SE*	*p*	*b*	*SE*	*p*
Treatment Duration (Months)	0.01	0.02	.712	−0.01	0.05	.956	−0.03	0.03	.297
% Females	−0.15	0.61	.801	0.08	0.65	.906	0.27	0.54	.621
*R*^2^ (*Q*)	0.00 (0.21)			0.00 (0.01)			0.00 (1.52)		

*n* number of studies, *b* unstandardized regression coefficient, *SE* Standard error of unstandardized regression coefficient. *R*^*2*^ Heterogeneity accounted for by predictors

### Association between self-harm measure timeframe and treatment efficacy

**Fig. 5:**
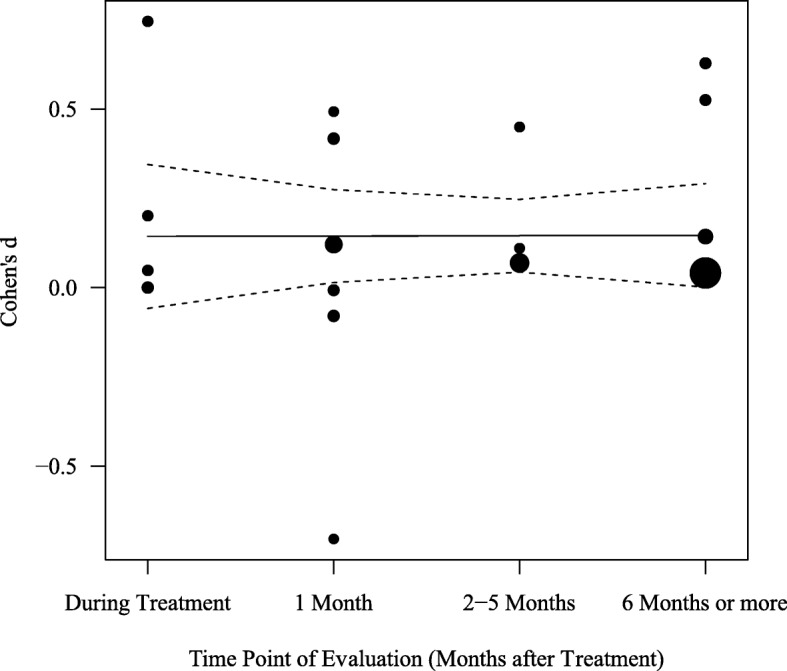
Effect sizes of the difference between therapeutic interventions and control groups by the time of measurement in self-harm. *Note.* The radius of the points is drawn proportional to the inverse of the standard errors (i.e., studies with greater statistical power are shown as larger points)

Given that the frequency of self-harm may fluctuate considerably across time, the timeframe in which each study measured self-harm is another study characteristic that may be associated with therapeutic treatment efficacy. After weighting for the sample size of each study (i.e., the inverse of the *SE*), correlation analysis found no evidence that measurement timeframe was associated with the efficacy of therapeutic interventions (*r* = .17, *p* = .533; see Fig. 5). This suggests that the magnitude of the difference between therapeutic interventions and active controls in reducing self-harm behaviour did not differ by the timeframe in which self-harm was measured.

### Publication Bias

**Fig. 6:**
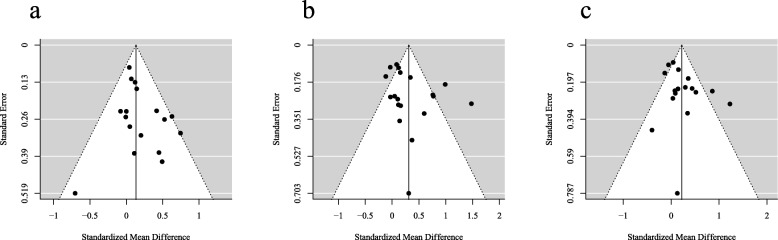
Funnel plots for (**a**) self-harm, **b** suicidal ideation and (**c**) depressive symptoms, showing limited evidence for publication biases across the three outcomes

Finally, we consider the potential impact of publication bias in the literature examined in these meta-analyses. For each outcome, funnel plots showing each study plotted by study precision and result are presented in Fig. 6. Visual inspection of these funnel plots suggests that across all three outcomes, studies were symmetrically distributed. That is, we found no evidence for publication bias in either the self-harm, suicidal ideation, or depressive symptoms literature included in the present meta-analyses. In addition, Egger’s regression found no evidence for funnel plot asymmetry in the analyses of self-harm (*z* = 1.59, *p* = .112), suicidal ideation (*z* = 1.10, *p* = .273), or depressive symptoms (*z* = 1.40, *p* = .162). Given that no indication of publication bias was found, no adjustments were needed according to trim-and-fill analysis in all analyses.

## Discussion

The clinical significance and high prevalence of self-harm and suicidal ideation among adolescents necessitates the establishment of efficacious psychotherapeutic treatments for young people. Given that the literature on therapeutic interventions for adolescent self-harm has proliferated since the last quantitative review [57], we conducted a systematic review and meta-analysis of 25 studies (including 9 studies new to this meta-analysis) focusing on the therapeutic interventions of self-harm and suicidal ideation in adolescents. Given the high degree of overlap between self-harm, suicidal thoughts and behaviours, and depression [27, 55, 63], we also considered depressive symptoms as a secondary outcome. This review addresses the general effects of therapeutic interventions compared to control interventions, as well as specific therapy-related effect-sizes through subgroup analysis. The comparison between several subgroups of therapeutic interventions allows a more nuanced understanding of the differential effects of treatment type in reducing self-harm, suicidal ideation, and depressive symptoms. Moreover, we assessed whether study characteristics such as self-harm measurement timeframe and gender distribution influenced the meta-analytic effect sizes of therapeutic intervention.

Our results indicate that compared to active control conditions, participants assigned to therapeutic interventions showed significantly greater decreases in self-harm behaviour (*d* = 0.13), suicidal ideation (*d* = 0.31), and depressive symptoms (*d* = 0.22) with small effect sizes. This is in line with former reviews on this topic [20, 21, 57], which also reported on the efficacy of therapeutic interventions in reducing self-harm. However, an overall analysis of participants assigned to exemplary models of routine clinical care (TAU, EUC) as control interventions also showed medium to large effects regarding the reductions in self-harm behaviour (*d* = 0.60), suicidal ideation (*d* = 0.87), and depressive symptoms (*d* = 0.51).

### Applicability of the results

#### Dialectic Behavioural therapy for adolescents

Subgroup analysis of specific types of therapeutic intervention revealed that, when compared to active control interventions, only DBT-A showed significantly better treatment outcomes with a moderate effect (*d* = 0.51). No other types of therapeutic interventions showed improvements when compared to active control interventions. A similar meta-analytic efficacy (*d* = 0.32) has been reported in a review of the effect of DBT in reducing self-harm behaviours in adult samples [12]. Within adolescent samples, we found low heterogeneity in results across studies assessing the efficacy of DBT-A. In terms of therapeutic types most effective for decreasing suicidal ideation, DBT-A also showed a small effect size against control interventions (*d* = 0.48) with moderate heterogeneity in results, and is larger compared to results of the meta-analysis of adult samples (*d* = 0.23, [12]).

#### Family-centred therapies

In contrast to the pattern of results for self-harm behaviours, family-centred therapies also showed a moderate effect against controls in reducing suicidal ideation (*d* = 0.58). However, as a subgroup family-centred therapies demonstrated a large degree of heterogeneity in results across studies. Notably, the two studies using Attachment-Based Family Therapy [13, 14•] showed the largest effects in reducing suicidal ideation compared to controls. Other studies within this subgroup include a variety of other interventions like family-based CBT [3•], systemic therapy [11•] and resourceful adolescent-parent program [59•]. Thus, the high heterogeneity in results may reflect the variety of interventions subsumed within this subgroup. Unfortunately, this variety limits our ability to draw reliable conclusions for the efficacy of this subgroup of therapeutic interventions, and so further research is needed to better evaluate family-centred therapies.

#### Promising therapies for future research

Additionally, two single studies were not assigned to a specific therapy subgroup but showed promising results with moderate to large effect sizes. A study focusing on MBT-A showed good efficacy in reducing self-harm behaviours, but also reported a high number of dropouts during the 12 months of treatment [64•]. Further, one study found a large effect of interpersonal therapy in reducing suicidal ideation and depressive symptoms [68•]. The promise of these two therapeutic interventions should be further tested in additional samples in order to better evaluate their efficacy in reducing self-harm behaviours, suicidal ideation, and depressive symptoms among adolescents.

### Pragmatic considerations

Given that therapeutic treatment for adolescent self-harm and suicidal ideation occurs in a variety of settings (e.g., inpatient clinics, outpatient care, and schools) which vary in the extent of available resources, we next take a pragmatic approach in considering the different therapy types. Both DBT-A and family-centred therapies were found to demonstrate efficacy in reducing suicidal ideation, with DBT-A also showing efficacy in reducing self-harm behaviours. Eligibility for recruitment into family-centred therapy studies was limited to young people with a primary caregiver who was also willing to participate (e.g., [11, 13, 14•]). Given that young people with a poorer quality of relationship with their family or who experience family stressors are at greater risk of engaging in self-harm [34, 36, 70], family-centred therapies have the potential to reduce family stressors and target behaviour of the young patients and their parents on a systemic level. However, in other cases therapeutic engagement with a primary caregiver may not be beneficial or should be carried out with caution (e.g., in cases of neglect, abuse by a caregiver). Thus, in deciding which therapeutic treatment to implement, consideration of the evidence-base as well as the resources available and the characteristics of the patients should be made.

### The possible benefits of treatment as usual

Across studies included in this meta-analysis, there seems to be an emerging pattern of evidence that TAU or EUC control conditions have the potential to reduce treatment outcomes (weighted pre-post effect sizes for self-harm: *d* = 0.60, for suicidal ideation: *d* = 0.87, for depressive symptoms: *d* = 0.51). In particular, experimental conditions only showed a small (although significant) improvement in reducing self-harm over-and-above control interventions. However, these findings should be interpreted with caution, as they may simply result from the time or environmental changes.

An alternative interpretation for this pattern of results is that a high standard of routine clinical care reduces self-harm behaviour, suicidal ideation, and depressive symptoms. In some studies, the reduction of self-harm was achieved faster in experimental conditions, such as CBT [34, 36] or DBT-A [47•], but by the endpoint of the study the experimental and active control conditions this difference was attenuated. Given the limited resources available in the field of child and adolescent psychotherapy and psychiatry, it is crucial to understand the mechanisms of change in the control interventions. One hypothesis is that recent years have seen interventions such as skills training and distress tolerance adapted from scientific research of treatment-pathways into routine clinical care, thus raising the efficacy of TAU conditions. Alternatively, it might be the case that adolescents engaging in self-harm behaviours benefit from regular therapeutic contact, regardless of whether this contact follows a pre-defined treatment pathway. In line with this hypothesis, routine clinical care has been demonstrated to be effective in treating adolescent depression [22]. In addition, this finding further underlines the results reported for general psychiatric management in adults with Borderline Personality Disorder (BPD), in which a comparable decrease of self-harm and BPD features have been reported from RCTs compared to evidence based treatment standards [25, 48, 49]. Understanding the factors which influence treatment outcome in TAU conditions to further optimize and standardise routine care is of the utmost importance given both the high prevalence of self-harm in adolescence and the restricted availability of therapists trained in special treatment approaches such as DBT-A. Consideration of both of these factors highlights the need for a scalable and easily teachable intervention to address the needs of a larger population.

### Limitations

The generalisability and nuance of the current meta-analyses are limited to a small, but growing, number of studies (*n =* 25 studies eligible for quantitative review). In particular, subgroup analyses were limited by the number of studies within each analysis, with some subgroups only containing one study (e.g., CBT in self-harm, [34, 36]). Thus, these treatment effects should be interpreted with caution, and future trials are needed in order to draw better conclusions in future reviews. Furthermore, the overall analysis revealed high heterogeneity of effects in suicidal ideation as well as depressive symptoms. However, the effects of all studies regarding self-harm showed only small heterogeneity. Another limitation concerns the demographic characteristics of the participants included in these studies. Across studies, young women tend to make up the majority of participants, with only one study in which young men made up 40% or more of the sample [2•]. In addition, despite growing evidence that transgender and gender-diverse young people have elevated rates of self-harm and suicidality compared to their cisgender peers [43, 69], none of the 25 trials included in this meta-analysis reported including any transgender or gender-diverse participants, severely curtailing our ability to assess the efficacy of either routine clinical care or psychotherapeutic treatment in a population with high-levels of clinical need.

### Future directions

Developmental differences in treatment efficacy also represent an area for future research. Although all studies included in the current meta-analyses included participants ranging from young adolescents (12–13 years old) to older adolescents (16–19 years old), no study reported outcomes stratified by age, limiting our ability to understand whether therapeutic efficacy differs across adolescent development. In addition to age-related differences in self-harm behaviour [60], key therapeutic targets such as emotion regulation skills change during early to late adolescence [24, 50]. Thus, future research should include age comparisons to consider not just *which* treatment intervention is most effective, but *when*.

Another important area for future research is to determine the optimal treatment setting for young people with severe self-harm. Recent studies have shown that longer inpatient admissions might be linked with increased risk of self-harm in young people [58, 62]. However, the role of hospital treatment, if any, remains to be established in young people with the greatest risk of suicide. Additionally, a standardized definition of what constitutes a recovery from self-harm behaviour (for discussion, see [37]) would increase the ability to compare across studies, as well as help to implement best-practice standards for evaluating the efficacy of therapeutic intervention.

Finally, given that only a minority of all studies reported data for follow-up assessments (e.g., [52]), little is known about whether the effect of therapeutic interventions in reducing self-harm remains stable over time. Follow-up analyses are of particular importance given preliminary evidence that reductions in self-harm are achieved faster by CBT [34, 36] or DBT-A [47•] than control intervention, but that this difference is attenuated at follow-up [34, 36, 47•]. The stability of therapeutic effects is an important factor in treatment considerations, particularly in resource-poor environments, and future follow-up research is needed to inform this decision-making process.

## Conclusion

The current meta-analysis with an overall sample of 2962 patients found small effect sizes for the reduction of self-harm and suicidal ideation in DBT-A, as well as moderate effect sizes for the reduction of suicidal ideation in family-centred therapy. Given that there is evidence for an increase in rates of self-harm through adolescence with a decline in early adulthood [19, 60], future studies should investigate age-specific effects of therapeutic interventions from young adolescents to early adulthood. Indeed, therapeutic interventions to treat self-harm and suicidal ideations for adolescents appear effective, but more research is needed to investigate the efficacy and effectiveness of specific therapeutic interventions.

Summing up the results from the last two decades of investigation into therapeutic interventions for self-harm and suicidal ideation, we conclude that besides DBT-A and family-centred therapy showing small-to-moderate effects, most groups of interventions have similar treatment outcomes to active controls. Nevertheless, control interventions (e.g., TAU, EUC) show large effect sizes in reducing self-harm, suicidal ideation, and depressive symptoms. These findings warrant a closer look at TAU treatment standards in clinical care as these standards may include useful components for decreasing self-harm. The efficacy of DBT-A and family-centred therapy (in particular, ABFT) underline the importance for more investigation into the additional benefits from these therapeutic interventions for self-harm and suicidal ideation in the future.

## Data Availability

Data are available from the original manuscripts cited in this meta-analysis.

## Footnotes

1 Three studies [40, 65, 66] were omitted due to missing baseline values.
